# Axitinib after Sunitinib in Metastatic Renal Cancer: Preliminary Results from Italian “Real-World” SAX Study

**DOI:** 10.3389/fphar.2016.00331

**Published:** 2016-09-28

**Authors:** Carmine D'Aniello, Maria G. Vitale, Azzurra Farnesi, Lorenzo Calvetti, Maria M. Laterza, Carla Cavaliere, Chiara Della Pepa, Vincenza Conteduca, Anna Crispo, Ferdinando De Vita, Francesco Grillone, Enrico Ricevuto, Michele De Tursi, Rocco De Vivo, Marilena Di Napoli, Sabrina C. Cecere, Gelsomina Iovane, Alfonso Amore, Raffaele Piscitelli, Giuseppe Quarto, Salvatore Pisconti, Gennaro Ciliberto, Piera Maiolino, Paolo Muto, Sisto Perdonà, Massimiliano Berretta, Emanuele Naglieri, Luca Galli, Giacomo Cartenì, Ugo De Giorgi, Sandro Pignata, Gaetano Facchini, Sabrina Rossetti

**Affiliations:** ^1^Oncology Unit, A.O.R.N. dei COLLI “Ospedali Monaldi-Cotugno-CTO,”Naples, Italy; ^2^UOSC Oncologia Cardarelli HospitalNaples, Italy; ^3^Oncology Unit 2, University Hospital of PisaPisa, Italy; ^4^Ospedale San BortoloVicenza, Italy; ^5^Division of Medical Oncology, Department of Internal and Experimental Medicine “F. Magrassi,” Second University of Naples – School of MedicineNaples, Italy; ^6^Department of Onco-Hematology Medical Oncology, S.G. Moscati Hospital of TarantoTaranto, Italy; ^7^Department of Uro-Gynaecological Oncology, Division of Medical Oncology, Istituto Nazional Tumori IRCCS “Fondazione G. Pascale,”Naples, Italy; ^8^Department of Medical Oncology, Istituto Scientifico Romagnolo per lo Studio e la Cura dei Tumori IRCCSMeldola, Italy; ^9^Unit of Epidemiology, Struttura Complessa di Statistica Medica, Biometria e Bioinformatica, Istituto Nazionale Tumori IRCCS “Fondazione G. Pascale,”Naples, Italy; ^10^Medical Oncology Unit, Azienda Ospedaliera “Mater Domini,”Catanzaro, Italy; ^11^Oncology Network ASL1 Abruzzo, Oncology Territorial Care Unit, Division of Medical Oncology, Department of Biotechnological and Applied Clinical Sciences, University of L'AquilaL'Aquila, Italy; ^12^Department of Medical, Oral and Biotechnological Sciences, University “G. D'Annunzio,”Chieti, Italy; ^13^Hepatobiliary Unit, Division of Abdominal Surgical Oncology, National Cancer Institute “G. Pascale Foundation,” IRCCSNaples, Italy; ^14^Pharmacy Unit, Istituto Nazionale Tumori IRCCS “Fondazione G. Pascale,”Naples, Italy; ^15^Division of Urology, Department of Uro-Gynaecological Oncology, Istituto Nazionale Tumori IRCCS “Fondazione G. Pascale,”Naples, Italy; ^16^Scientific Direction, Istituto Nazionale Tumori IRCCS “Fondazione G. Pascale,”Naples, Italy; ^17^Division of Radiation Oncology, Istituto Nazionale Tumori IRCCS “Fondazione G. Pascale,”Naples, Italy; ^18^Division of Medical Oncology, CROAviano, Italy; ^19^Division of Medical Oncology, Istituto Oncologico Giovanni Paolo IIBari, Italy

**Keywords:** mRCC, first-line treatment, axitinib, real-life patient, mPFS

## Abstract

Axitinib is an oral angiogenesis inhibitor, currently approved for treatment of metastatic renal cell carcinoma (mRCC) after failure of prior treatment with Sunitinib or cytokine. The present study is an Italian Multi-Institutional Retrospective Analysis that evaluated the outcomes of Axitinib, in second-line treatment of mRCC. The medical records of 62 patients treated with Axitinib, were retrospectively reviewed. The Progression Free Survival (PFS), the Overall Survival (OS), the Objective Response Rate (ORR), the Disease Control Rate (DCR), and the safety profile of axitinib and sunitinib–axitinib sequence, were the primary endpoint. The mPFS was 5.83 months (95% CI 3.93–7.73 months). When patients was stratified by Heng score, mPFS was 5.73, 5.83, 10.03 months according to poor, intermediate, and favorable risk group, respectively. The mOS from the start of axitinib was 13.3 months (95% CI 8.6–17.9 months); the observed ORR and DCR were 25 and 71%, respectively. When stratified patients by subgroups defined by duration of prior therapy with Sunitinib (≤ vs. >median duration), there was a statistically significant difference in mPFS with 8.9 (95% CI 4.39–13.40 months) vs. 5.46 months (95% CI 4.04–6.88 months) for patients with a median duration of Sunitinib >13.2 months. DCR and ORR to previous Sunitinib treatment was associated with longer statistically mPFS, 7.23 (95% CI 3.95–10.51 months, *p* = 0.01) and 8.67 (95% CI 4.0–13.33 months, *p* = 0.008) vs. 2.97 (95% CI 0.65–5.27 months, *p* = 0.01) and 2.97 months (95% CI 0.66–5.28 months, *p* = 0.01), respectively. Overall Axitinib at standard schedule of 5 mg bid, was well-tolerated. The most common adverse events of all grades were fatig (25.6%), hypertension (22.6%), gastro-intestinal disorders (25.9%), and hypothyroidism (16.1%). The sequence Sunitinib–Axitinib was well-tolerated without worsening in side effects, with a median OS of 34.7 months (95% CI 18.4–51.0 months). Our results are consistent with the available literature; this retrospective analysis confirms that Axitinib is effective and safe in routine clinical practice.

## Introduction

Renal Cell Carcinoma (RCC) accounts for 2–3% of all adult malignancies, representing the seventh most common cancer in men and the ninth most common cancer in women (Escudier et al., 2013). Its incidence has increased over the past several years, contributing to increasing mortality rate. RCC is a family of tumors, each with distinct genetic landscapes, different growth patterns, and metastatic potentials, resulting in a heterogeneous group of disease processes (Linehan et al., 2013). Clear cell renal cell carcinoma (ccRCC) is the most common RCC (75–80% of all primary kidney malignancies; Cohen and McGovern, 2005). Approximately 25–30% of patients with kidney cancer present metastases at the time of diagnosis and ~30% develop recurrent or metastatic disease after radical treatment for localized disease (Leung et al., 2014). Recurrent and/or metastatic renal cell carcinoma (mRCC) is associated with a poor 5-years survival, roughly 10%, however, in the last decade, a series of novel agents have been introduced in clinical practice and the outcomes are slowly improving. Angiogenesis plays a central role in the development, growth, and metastatic progression of RCC via VHL, HIF 1α, VEGF, PDGF, mTOR (PI3K/AKT signaling; Nicol et al., 1997; Dorević et al., 2009; Kornakiewicz et al., 2013; Dimova et al., 2014). Despite an increasing knowledge about the genetic and signaling abnormalities involved in the RCC carcinogenesis, such as VHL, PBRM1, SETD2, and BAP1, no biomarkers are currently available thus there are no molecular factors which may guide the clinicians in choosing the therapeutic strategy (Brugarolas, 2014; D'Aniello et al., 2014; Cavaliere et al., 2015). The advent of the Target Therapies (TTs) has revolutionized the mRCC treatment with an impressive effect on the Overall Survival (OS), which increased from an average of 9 months in 1995, when the only option in first line was interferon-alfa (IFN-α), to a median of 28–29 months in 2013, the TTs era (Motzer and Russo, 2000; Chowdhury et al., 2008; Albiges et al., 2015; de Velasco et al., 2015). The TTs include the Tyrosine Kinase Inhibitors, TKIs, targeting the Vascular Endothelial Growth Factor Receptors, VEGFRs, the mammalian Target of Rapamycin inhibitors, mTORis, and Bevacizumab, a monoclonal antibody targeting the VEGF ligand.

Axitinib, a potent oral and selective TKi of VEGFR-1, VEGFR-2, and VEGFR-3, has been recently approved in second line after sunitinib or cytokines failure. The second-line phase III AXIS trials compared axitinib, 5 mg twice daily dose-titrated up or down to tolerance, with sorafenib in patients who progressed despite first-line therapy containing sunitinib, bevacizumab plus IFN-α, temsirolimus, or cytokines. The median Progression Free Survival (mPFS), primary endpoint, was 6.7 months with axitinib compared to 4.7 months with sorafenib (HR: 0.665; 95% CI 0.544–0.812; one-sided *p* < 0.0001) regardless of prior treatment. Partial responses were seen more often after Axitinib than Sorafenib (19.4 vs. 9.4%, *p* < 0.001). In patients previously treated with Sunitinib, mPFS was 4.8 months in Axitinib arm and 3.4 months in Sorafenib arm (*p* = 0.011; Rini et al., 2011). In a recent update, the mOS was 20.1 (95% CI 16.7–23.4) with Axitinib and 19.2 months (17.5–22.3) with Sorafenib (HR: 0.969, 95% CI 0.800–1.174; one-sided *p* = 0.3744; Hutson et al., 2013; Motzer et al., 2013). The most common adverse events were diarrhea, hypertension, and fatig. To date, according to National Cancer Guidelines, Axitinib, Everolimus, and Sorafenib are registered in second-line treatment of mRCC. Evidences from randomized clinical trials, retrospective studies or single-institution experiences do not provide clear and conclusive information which might guide the clinician in choosing Axitinib rather than Everolimus than Sorafenib, or vice versa, in the second-line setting, hence the decision is made exclusively on the basis of the safety profile and patients medical history. Several “real world” studies have showed the efficacy and safety of Axitinb in unselected populations (Vogl et al., 2013; Basso et al., 2014; Maroto et al., 2014; Matias et al., 2014; Signorovitch et al., 2015; Vogelzang et al., 2015, 2016; Guida et al., 2016; Hutson et al., 2016; Laskey et al., 2016; Pal et al., 2016; Wagstaff et al., 2016), we thought to further reinforce such evidences publishing our own experience with the drug.

## Patients and methods

This is a multi-institutional, observational, retrospective study (SAX), which was carried out in nine Italian Oncology Centers, after approval by the National Cancer Institute of Naples Institutional Board. Medical records of patients who were treated with axitinib, in second line, between January 2014 and January 2016 were retrospectively reviewed. All subjects gave written informed consent in accordance with the Declaration of Helsinki. To be eligible, patients were required to meet the following inclusion criteria: aged ≥18 years, histologically confirmed RCC, treatment with Axitinib started between January 2014 and January 2016 with at least one radiological reassessment of disease, radiologically measurable disease according to RECIST 1.1 criteria, first line treatment with Sunitinib at least 2 months of therapy. We administered Axitinib according to the conventional and dose-titration schedule.

The primary endpoint was Progression Free Survival, PFS, Overall Survival, OS, Objective Response Rate, ORR, Disease Control Rate, DCR, and the safety profile of Axitinib and Sunitinib–Axitinib sequence. The secondary objectives included the potential relationships between Patient's demographics and baseline characteristics, AEs and response to treatment. PFS was defined as the interval between the date of the first dose of Axitinib and the date of the disease progression or death from any cause; disease progression was defined as radiological tumor progression according to Response Evaluation Criteria In Solid Tumors, RECIST, version 1.1, or clinical progression, including death. AEs were graded according to Common Terminology Criteria for Adverse Events version 4.0.

Descriptive statistics were used to describe Patient's demographics and baseline characteristics, treatment patterns and Adverse Events, AEs. Categorical variables are described by patient counts and percentages. The univariate risks of progression and death were examined: PFS by treatment line of therapy and OS curves were estimated by the Kaplan–Meier and selected variables were compared using two-sided log-rank test. The Cox proportional hazards model was fitted to estimate the risk of progression or of death; hazard ratio (HR) and 95% CI was estimated, adjusted for age, gender, and center. The SPSS statistical package version 23.0 (SPSS Inc., Chicago, IL) was used for all statistical analysis.

## Results

Between Jan 2014 and Jan 2016, 62 patients with mRCC or recurrent RCC were eligible for the final analysis.

Patient's demographics and baseline characteristics were summarized in Table 1. Median age was 62 years old, most patients were male (88.7%), the 67.7% had an ECOG Performance Score of 0. Of the 62 patients, 54 (87.1%) had prior nephrectomy and only 4.8% (3/62) had a histological diagnosis different from clear cell. Lung was the first site of metastasis (47.8%) and 6.5% (4/62) of patients had liver metastasis. Based on the MSKCC score classification, only 6.5% (4/62) patients were “poor risk,” the most being “intermediate” 69.4% and “good” 24.2%, while according to Heng score, 12.9% (8/62) patients were “poor risk,” 67.7 and 19.4% were intermediate and favorable risk, respectively.

**Table 1:** **Baseline demographic and clinical patients' characteristics**.

**Baseline characteristics**	***N***	**(%)**
Median age years (range)	62	(36–86)
Age		
≤ 65	39	(62.9%)
>65	23	(37.1%)
Gender		
Male	55	(88.7%)
Female	7	(11.3%)
ECOG PS		
0	42	(67.7%)
1	18	(29.0%)
2	2	(3.2%)
Prior nephrectomy		
Yes	54	(87.1%)
No	8	(12.9%)
MOTZER score		
Poor	4	(6.5%)
Intermediate	43	(69.4%)
Favorable	15	(24.2%)
HENG score		
Poor	8	(12.9%)
Intermediate	42	(67.7%)
Favorable	12	(19.4%)
Site of disease		
Lung	29	(47.8%)
Bone	8	(12.9%)
Liver	4	(6.5%)
Lymph node	9	(14.5%)
Other	12	(19.4%)

### Clinical outcomes

**Figure 1:**
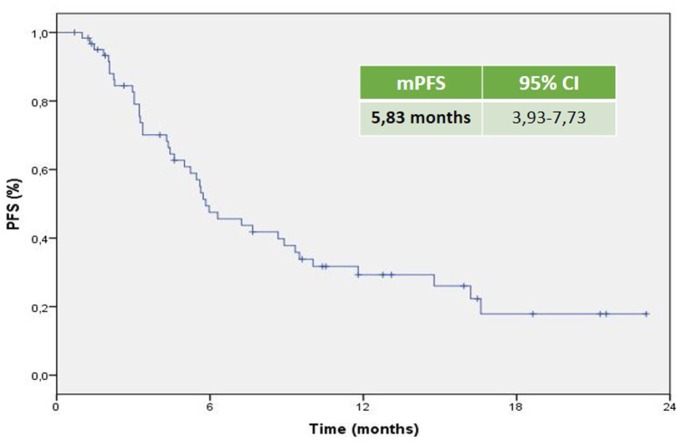
**Kaplan–Meier curve of median PFS of the patients under study**.

**Figure 2:**
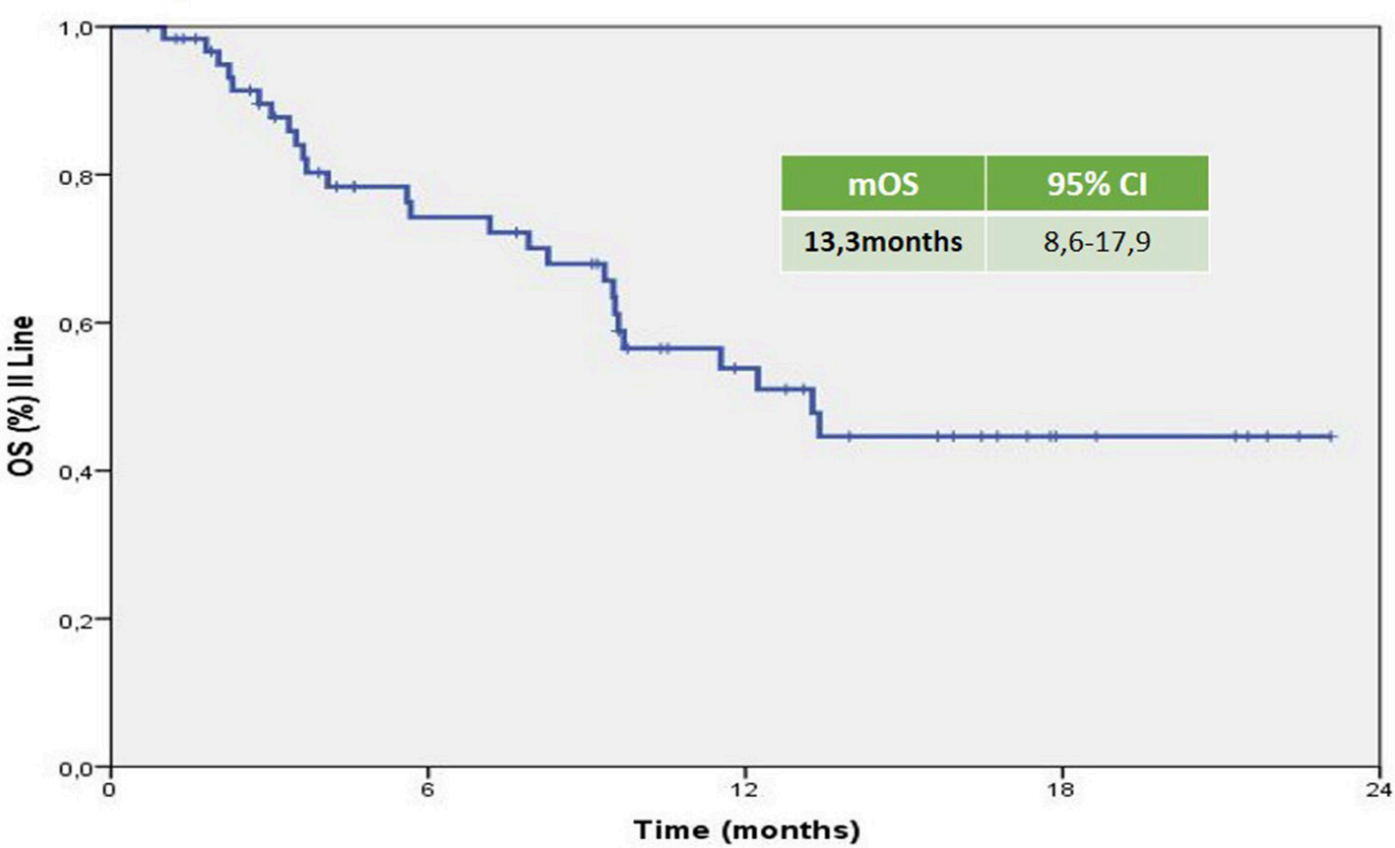
**Kaplan–Meier curve of median OS of the patients under study from the start of axitinib**.

**Figure 3:**
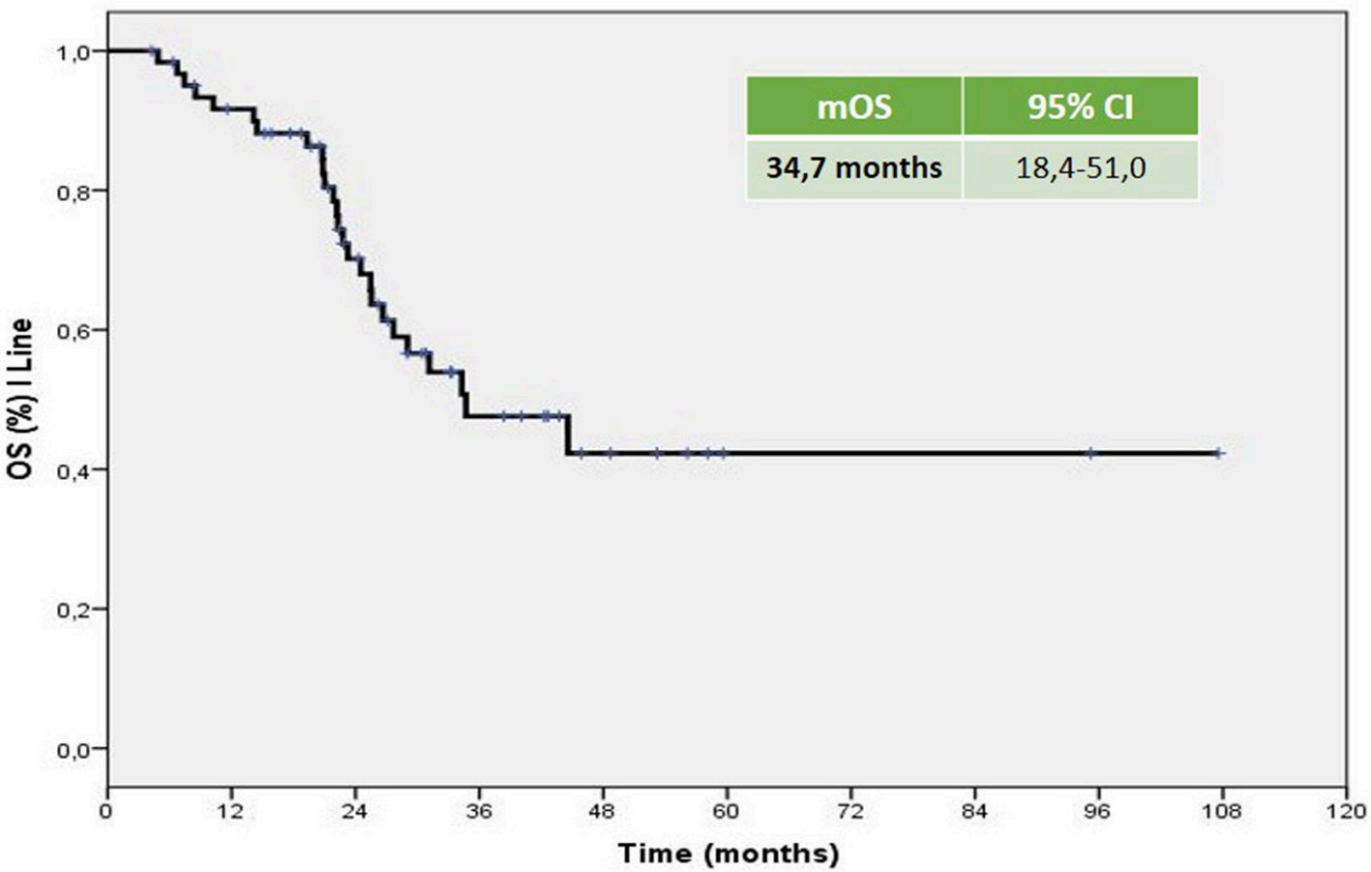
**Kaplan–Meier curve of overall median OS of the patients under study, sunitinib–axitinib sequence**.

**Figure 4:**
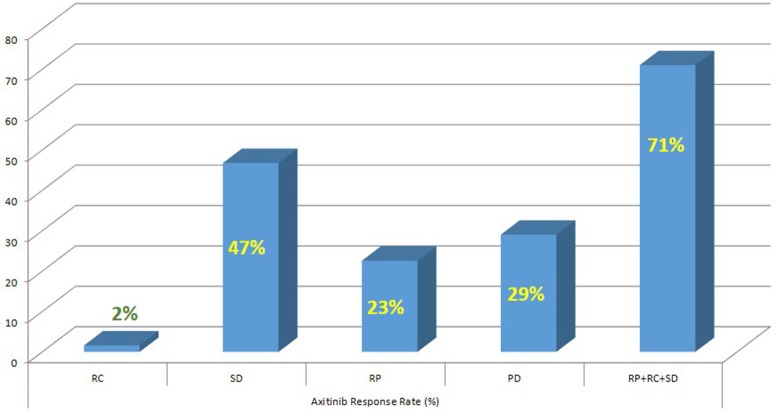
**Axitinib response rate (%)**.

The mPFS was 5.83 months (95% CI 3.93–7.73 months; Figure 1). The mOS from the start of Axitinib, was 13.3 months (95% CI 8.6–17.9 months; Figure 2). The ORR, according to RECIST criteria version 1.1 (Eisenhauer et al., 2009) was 25%, with the 23% of patients achieving a Partial Response, PR, and one patient having a Complete Response, CR (Figure 3). The DCR to Axitinib was 71% and correlated with longer statistically mPFS, 9.33 (95% CI 5.51–13.15 months, *p* < 0.001) vs. 3.26 months (95% CI 2.98–3.54 months, *p* < 0.001), respectively. DCR and ORR to previous Sunitinib treatment was associated with longer statistically mPFS, 7.23 (95% CI 3.95–10.51 months, *p* = 0.01) and 8.67 (95% CI 4.0–13.33 months, *p* = 0.008) vs. 2.97 (95% CI 0.65–5.27 months, *p* = 0.01) and 2.97 months (95% CI 0.66–5.28 months, *p* = 0.01), respectively (Table 2). mOS was not reached for ORR (RC+RP), the mean OS according to DCR and ORR was 14.95 (95% CI 12.4–17.49 months, *p* = 0.056) and 15.66 (95% CI 12.19–19.10 months, *p* = 0.057) vs. 7.4 (95% CI 3.85–11.0 months, *p* = 0.056) and 7.42 (95% CI 3.81–11.02 months, *p* = 0.057), respectively (Table 2). When stratified patients by subgroups defined by duration of prior therapy with Sunitinib (≤ vs. >median duration), there was a statistically significant difference in mPFS with 8.9 (95% CI 4.39–13.40 months, *p* = 0.03) vs. 5.46 months (95% CI 4.04–6.88 months, *p* = 0.03) for patients with a median duration of Sunitinib >13.2 months without differences in mOS (*p* = 0.27; Table 2). No differences in terms of mPFS according to type of sunitinib-schedule (standard schedule vs. modified schedule; *p* = 0.6; Table 2). When patients was stratified by Heng score mPFS was 5.73, 5.83, 10.03 months according to poor, intermediate and favorable risk group, respectively. The same results was achieved when used Motzer score, but only Heng score stratification correlated statistically significantly with OS (*p* = 0.02; Table 2). There was no statistically significant difference in mPFS and mOS when stratified patients according to ECOG Performance Status (*p* = 0.8 and 0.7, respectively; Table 2). Prior nephrectomy correlated with longer statistically mPFS, 6.30 (95% CI 2.77–9.82 months, *p* = 0.01) vs. 3.03 months (95% CI 2.86–3.20 months, *p* = 0.01), respectively and also with longer statistically mOS (*p* = 0.003; Table 2), even if the median OS was not reached. Overall Axitinib at standard schedule of 5 mg bid, was well-tolerated, with no grade 4 toxicity. Dose reduction occurred in 21% (13/62) of which five patients in the titration group. The most common adverse events of all grades were fatig (25.6%) hypertension (22.6%), gastro-intestinal disorders (25.9%), hypothyroidism (16.1%), hand-foot syndrome (8.1%), we observed no cases of dysphonia (Table 3). According to AEs recorded, we did not find predictor of better outcome. The sequence Sunitinib–Axitinib was well-tolerated without worsening in side effects, particularly in terms of hypertension and hand–foot syndrome, with a mOS for the overall analysis population of 34.7 months (95% CI 18.4–51.0 months; Figure 4). Table 4 summarized the adjusted hazard ratios for PFS and OS according to prior nephrectomy, duration, ORR, and DCR of prior treatment with Sunitinib. The multivariate model was adjusted for age, gender, and center. Prior nephrectomy and DCR showed a significant independent impact either for PFS (HR: 0.32; 95% CI 0.13–0.78, *p* = 0.01; HR: 0.35; 95% CI 0.15–0.81, *p* = 0.01, respectively), and for OS (HR: 0.24; 95% CI 0.08–0.68, *p* = 0.007; HR: 0.36; 95% CI 0.13–0.98, *p* = 0.047, respectively). Duration of prior treatment (>13 months) and ORR showed a significant independent role only in the PFS (HR: 0.48; 95% CI 0.25–0.94, *p* = 0.03; HR: 0.28; 95% CI 0.10–0.77, *p* = 0.01, respectively).

**Table 2:** **Univariate analysis of mPFS and mOS in our study population**.

	***p*****-value**	
	**mPFS**	**mOS**
Tumor response rate to prior sunitinib		
DCR	0.01	0.056
ORR	0.008	0.057
Duration of prior sunitinib treatment		
≤ 13.2 vs. >13.2 months	0.03	0.27
Heng score	0.3	0.02
ECOG PS	0.8	0.7
Prior nefrectomy	0.01	0.003

**Table 3:** **Adverse events of axitinib in our study population**.

**AE**			**Axitinib titration**	
	**All grades (*n* = 62) *N* (%)**	**≥Grade 3 (*n* = 62)**	**All grades (*n* = 15) *N* (%)**	**≥Grade 3 (*n* = 15)**
Hematologic	2 (3.3)	–	–	–
Hypertension	11 (18)	3 (4.9)	2 (13.3)	1 (6.7)
Gastro-intestinal	9 (14.5)	3 (4.8)	2 (13.3)	–
Hypothyroidism	10 (16.1)	–	3(20)	–
Fatig	11 (17.7)	5(8.1)	3(20)	1 (6.7)
Hepatic	1 (1.6)	–	–	–
Hand–foot syndrome	4 (6.5)	1 (1.6)	2(13.3)	1(6.7)
Dysphonia	–	–	2(13.3)	–

**Table 4:** **Multivariate adjusted Cox model**.

	**Progression-free survival (PFS)**			**Overall survival (OS)**		
	HR^*^	**(95% CI)**	***p*-value**	HR^*^	**(95% CI)**	***p*-value**
**NEFRECTOMY**						
Yes	0.32	(0.13–0.78)	0.01	0.24	(0.08–0.68)	0.007
**PRIOR TREATMENT**						
(>13 months)	0.48	(0.25–0.94)	0.03	0.56	(0.24–1.30)	0.2
**ORR**						
(RC + RP)	0.28	(0.10–0.77)	0.01	0.42	(0.12–1.48)	0.2
**DCR**						
(RC + RP + SD)	0.35	(0.15–0.81)	0.01	0.36	(0.13–0.98)	0.047

HR, hazard ratio; 95% CI, 95% confidence interval.

* Multivariate Cox model adjusted for terms of age (continuous), gender, and center.

### Outcome in dose titration axitinib

Dose escalation to 7 or 10 mg bid was feasible in 15 patients (24.2%). mPFS was longer, but not statistically significant, than patients without dose titration, 10.03 (95% CI 2.05–18.015 months, *p* = 0.07) vs. 5.63 months (95% CI 4.92–6.34 months, *p* = 0.07), respectively (Table 2). mOS was not significant for dose titration too (*p* = 0.09, Table 2). Dose titration was well-tolerated with only increase in the incidence of hypothyroidism and fatig, no worsening of hypertension (Table 3).

## Discussion

The development of TTs, VEGFR-TKi, and mTORi, resulted in a significant improvement in terms of both PFS and OS. However, resistance to drugs will occur in most patients. A reasonable approach to overcome drug resistance is the use of sequential therapy, and some drugs that showed efficacy in the second-line setting are in use. The optimization of the disease management should be based on clinical trials but also on real experiences, also known as “real world” or “real life” studies, which are more reproducible compared with randomized trials.

Axitinib, a second-generation multi-targeted TKI against VEGFR1, 2 and 3, is licensed in Europe for the treatment of adult patients with mRCC after failure of Sunitinib or cytokines. Efficacy is based on the AXIS trial which reported a longer mPFS in Sunitinib pretreated patients of 6.5 months (95% CI 5.7–7.9 months) with a mOS of 15.2 months (95% CI 12.8–18.3 months). In our analysis, the mPFS was 5.83 months (95% CI 3.93–7.73 months) with mOS of 13.3 months (95% CI 8.6–17.9 months). These results are similar to the Axis trial and the other real-life experiences (Vogl et al., 2013; Basso et al., 2014; Maroto et al., 2014; Matias et al., 2014; Signorovitch et al., 2015; Vogelzang et al., 2015, 2016; Guida et al., 2016; Hutson et al., 2016; Laskey et al., 2016; Pal et al., 2016; Wagstaff et al., 2016). The majority of the evaluated studies are retrospective analysis of indirect comparison between standard second-line treatments (Everolimus vs. Axitinib) and use Axitinib in second or third line with the median duration of therapy as surrogate of mPFS; others are available only in abstract form, therefore not comparable. Evidences from randomized clinical trials, retrospective studies or single-institution experiences do not provide clear and conclusive information about the optimal sequencing of TTs, furthermore, there is no patients stratification based on prognostic criteria as in first-line. The IMDC model (Heng score) was recently validated in second-line setting resulting more reliable when compared with the three-factor MSKCC model (Heng et al., 2014; Ko et al., 2015). In our retrospective study, when the patients were stratified by Heng score, mPFS was 5.73, 5.83, 10.03 months according to poor, intermediate and favorable risk group, respectively. To date, sequential treatment with VEGF-TKIs or mTORi, represents the standard of care (Chowdhury et al., 2008; Facchini et al., 2009; Calvo et al., 2012; Rizzo et al., 2015; Ko et al., 2015; Linee guida^1^). The use of EVE is supported by the RECORD-1 trial though the population was not homogeneous for line of treatment (patients who have had two or more prior therapies were also included) and the RECORD-4, a phase II trial, including only patients in the second-line setting, confirmed the efficacy of EVE after SUN or other first-line therapies (Motzer et al., 2008; Motzer R. et al., 2015). The RECORD-4 mPFS was 5.7 months (95% CI 3.7–11.3) with previous Sunitinib, ORR was 7% (95% CI 2–17) and OS 23.8 months (95% CI 13.7-NE; Table 5). The activity of the sequence VEGF-TKI–VEGF-TKI has been demonstrated by several trials showing a longer statistically significant mPFS and in some mOS too (Rini et al., 2011; Motzer et al., 2013; Motzer R. et al., 2015; Hutson et al., 2014; Eichelberg et al., 2015). A network meta-analysis conducted by Leung et al. suggested that axitinib is a more suitable TT option, compared to sorafenib and pazopanib, after failure of first line treatment; Sunitinib and Axitinib seem associated with a superior clinical benefit when compared to Sorafenib, Pazopanib, and Temsirolimus; in particular Axitinib is associated with the lowest risk of withdrawal due to adverse events (Leung et al., 2014). Our analysis confirms these results. Many clinicians choose second-line TTs based on response to first-line therapy and depth of remission. Escudier et al. (2014) conducted a *post hoc* analysis of AXIS trial to evaluate the efficacy of Axitinib and Sorafenib by response to prior therapy, duration of prior therapy, and tumor burden in patients previously treated with Sunitinib or cytokines. In regards to the prior therapy response, they did not found statistically significant differences in PFS or OS in responders vs. non-responders. In regards to duration of prior therapy with Sunitinib, defined as shorter (< median) vs. longer (≥median), they detected a significantly longer PFS with second-line Axitinib only in patients who had received a longer prior cytokines treatment (no relationship in patients treated with Sunitinib neither in ones who had Sorafenib in second line); OS with second-line Axitinib or Sorafenib was significantly longer in patients who received longer prior therapy, except in those treated with Sunitinib followed by Axitinib (Escudier et al., 2014). On the contrary our analysis showed that, when stratified patients by subgroups defined by duration of prior therapy with Sunitinib (≤ vs. >median duration), there was a statistically significant difference in mPFS with 8.9 (95% CI 4.39–13.40 months, *p* = 0.03) vs. 5.46 months (95% CI 4.04–6.88 months, *p* = 0.03) for patients with a median duration of Sunitinib >13.2 months, without differences in mOS. DCR and ORR to previous Sunitinib treatment was associated with longer statistically mPFS, 7.23 and 8.67 vs. 2.97 and 2.97 months, respectively. mOS was not reached for ORR (RC+RP), the mean OS according to DCR and ORR was 14.95 and 15.66 vs. 7.4 and 7.42 months, respectively. Our results are consistent with those of Elaidi et al. who conducted a retrospective analysis of 241 m-ccRCC patients who received a first-line TKI for ≥6 months followed by a second-line TKI or mTORi, showing that patients who remained on first-line TKI between 11 and 22 months benefited from a TKI rechallenge rather than from second-line mTORi (PFS [HR≈0.5; median PFS (months): 9.4 (5.9–12.2) vs. 3.9 (3.0–5.5), *p* = 0.003; TTF(months): 8.0 (5.5–11.0) vs. 3.6 (3.0–4.6), *p* = 0.009]; Elaidi et al., 2015). Historically, VEGF-targeted therapy was reported to achieve higher ORR (20–30%) compared to mTOR-targeted therapy (≤ 10%), which is supported by our analysis (Grünwald et al., 2015). The lack of response to a VEGF-targeted agent in the first-line setting does not preclude positive clinical outcomes with a second-line VEGF-targeted agents. Prior nephrectomy correlated with longer statistically mPFS (*p* = 0.01) and mOS (*p* = 0.003). Overall Axitinib at standard schedule of 5 mg bid, was well-tolerated, with no grade 4 toxicity. The most common adverse events of all grades were fatig (25.6%), gastro-intestinal disorders (25.9%), hypertension (22.6%), and hypothyroidism (16.1%). Our data showed a lower incidence of AEs than AXIS trial. According to AEs recorded, we did not find predictor of better outcome, unlike the AXIS analysis showed that patients who developed diastolic blood pressure ≥90 mmHg or systolic below 140 mmHg within the first 8 or 12 weeks of randomization had longer mOS. Dose escalation to 7 or 10 mg bid was feasible in 15 patients (24.2%), without statistically significant differences in terms of both mPFS and OS. Dose titration was well-tolerated with only increase in the incidence of hypothyroidism and fatig, no worsening of hypertension. It was interesting to note that the 19.3% (12/62) of patients enrolled in our analysis used a modified schedule of Sunitinib (2 w on 1 w off) in first line treatment without differences in terms of outcomes. These results, although on a small size population, confirm those of several retrospective study that evaluated Sunitinib administered on alternative schedules with reduction in the AEs and achieving comparable outcomes to the standard schedule (Atkinson et al., 2014; Bracarda et al., 2015; Lee et al., 2015). Our analysis confirm that sequence TKI-TKI (Sunitinib–Axitinib) was well-tolerated without worsening in side effects, with a mOS for the overall analysis population of 34.7 months (95% CI 18.4–51.0 months), consistent with those of the AXIS trial (33.7 months, 95%CI 28.6–36.96 months). Recently were published the results of CheckMate 025 in which Nivolumab, a programmed death 1 (PD-1) checkpoint inhibitor, was associated with longer mOS, 25.0 months (95% confidence interval [CI], 21.8 to not estimable) vs. 19.6 months (95% CI, 17.6 to 23.1) with everolimus, in previous treated mRCC patients with one or two regimens of anti-angiogenic therapy. It will be interesting to evaluate the efficacy of nivolumab vs. VEGFRi, such as axitinib in second pure line of treatment and their sequencing (Motzer R. J. et al., 2015).

**Table 5:** **Comparison of mPFS, mOS, and tumor response between RECORD 4 and SAX population**.

	**RECORD 4 (*n* = 58)**	**SAX (*n* = 62)**
mPFS (mo)	5.7	5.83
mOS (mo)	23.8	13.3
DCR (%)	41	71
ORR (%)	7	25
Best overall response (%)		
RC	1	2
PR	4	23
SD	37	47
PD	15	29

n, number of patients; CR, complete response; PR, partial response; SD, stable disease; PD, progressive disease; ORR, overall response rate; DCR, disease control rate.

## Conclusions

Our results are consistent with the available literature; this retrospective analysis confirms the efficacy and safety of Axitinib in an unselected population. DCR, ORR, duration of prior therapy with Sunitinib, and prior nephrectomy may represent prognostic factor of a longer mPFS with Axitinib while only DCR and prior nephrectomy correlated with a longer mOS.

## Author contributions

CDA, MV, AF, GF, and SR collected data and wrote the manuscript; AC performed the statistical analysis; LC, ML, CC, CDP, VC, FD, FG, ER, MDT, RD, MDN, SC, GI, AA, RP, GQ, SPisconti, GCiliberto, PMaiolin, PMuto, SPerdonà, MB, LG, GCartenì, UD, and S Pignata performed the data collection and the elaboration of data.

### Conflict of interest statement

The authors declare that the research was conducted in the absence of any commercial or financial relationships that could be construed as a potential conflict of interest.

## Footnotes

^1^Linee guida. *Linee guida AIOM*. Available online at: www.aiom.it
